# *Metapocyrtuskitangladensis* sp. n., a new *Pachyrhynchuscumingii* GR Waterhouse, 1841 mimic from Mindanao Island, Philippines

**DOI:** 10.3897/zookeys.853.30595

**Published:** 2019-06-06

**Authors:** Analyn A. Cabras, Milton Norman D. Medina, Guanyang Zhang

**Affiliations:** 1 Coleoptera Research Center, Institute for Biodiversity and Environment, University of Mindanao, Davao City, 8000, Philippines University of Mindanao Davao Philippines; 2 School of Life Sciences, Arizona State University, Tempe, AZ 85287, USA Arizona State University Tempe United States of America

**Keywords:** Bukidnon, endemism, Mt Kitanglad Range Natural Park, weevil

## Abstract

A description of a new species from the genus *Metapocyrtus* Heller, 1912 (Coleoptera: Curculionidae: Pachyrhynchini) from Mt Kitanglad Range Natural Park, an ASEAN Heritage Site in northern Mindanao is presented and illustrated. The recent discovery is also the first mimic record of *Pachyrhynchuscumingii* GR Waterhouse, 1841 which are both found in the same locality. A *Coptorhynchus* sp. showing similar elytral patterns was also documented to be part of the mimicry complex. The new species differs from the other two species in having a distinct transverse groove between forehead and rostrum and the antennal scape reaching beyond the hind margin of the eye.

## Introduction

The province of Bukidnon is one of the most entomologically explored areas in Mindanao Island, Philippines, with dozens of species of Pachyrhynchini Schönherr, 1826 (Coleoptera: Curculionidae) discovered between 20^th^ and 21^st^ centuries (Schultze 1923, 1925; Bollino et al. 2017; Rukmane and Barševskis 2016; Cabras and Barševskis 2016). The province name means “highlander” or “mountain dweller” and its topography is rugged with numerous mountain ecosystems. One of the known mountain ecosystems in Bukidnon is Mt Kitanglad Range Natural Park (MKRNP), a 47,270-hectare ASEAN heritage park (DENR 2018) located in northern part of Bukidnon. It is one of the key biodiversity areas in the Philippines (Canoy and Suminguit 2001) and considered as an important bird area for it is the home of the Philippine Eagle (*Pithecophagajefferyi* Ogilvie-Grant, 1897), the Philippine National Bird. Although several biodiversity expeditions have been conducted in MKRNP (Peterson et al. 2008; Tan et al. 2015; Rickart et al. 2003; James 2004; Naïve 2017; Cabactulan et al. 2017), no Coleoptera expedition was conducted until recently.

Part of the vision of the Coleoptera Research Center of the University of Mindanao Davao City is to document the coleopteran fauna of the different mountain ecosystems in Mindanao. In partnership with various stakeholders in Bukidnon, a Coleoptera expedition was conducted in Barangay Chinchona, Lantapan, which is one of the main trails of MKRNP. One of the interesting species belonging to the genus *Metapocyrtus* Heller, 1912 was discovered and further examination revealed it as a species new to science. The new species herein together with data on its ecology, distribution, and mimicry with *Pachyrhynchuscumingii* is described and illustrated. Mimicry among the tribe Pachyrhynchini has been widely recorded (Barševskis 2014, 2016, 2017; Cabigas 2010; Vives 2013) since the time of Wallace (1889) and Schultze (1923); however, we had barely scratched the surface of this topic considering so many mimics await discovery and description.

## Materials and methods

The specimens deposited in the University of Mindanao Coleoptera Research Center (**UMCRC**) were collected through sheet beating and hand picking and killed in vials with ethyl acetate. Morphological characters were observed under Luxeo 4D and Nikon SMZ745T stereomicroscopes. Stacked digital habitus images were taken with Nikon D5300 digital camera and Sigma 18–250 macro lens, whereas digital images of genitalia were taken with Ricoh WG-50. All images were stacked and processed using a licensed version of the software Photoshop CS6 Portable. Endophallus eversion was done by Dr Bollino and images were taken with Nikon D90 digital camera, extension tubes, bellows, and Rodenstock Rodagon 60mm f/5.6 lens. Images were then stacked and processed using a licensed version of the software Helicon Focus 6.7.0. Measurements mentioned in this paper are abbreviated as follows:

**LB** body length, from the apical margin of pronotum to the apex of elytra;

**LE** elytral length, from the level of the basal margins to the apex of elytra;

**WE** maximum width across the elytra;

**LP** pronotal length, from the base to apex along the midline;

**WP** maximum width across the pronotum;

**LR** length of rostrum;

**WR** maximum width across the rostrum.

All measurements are given in millimeters and follow the measurement methodology of Yoshitake (2013). The specimens are deposited in the following collections:

**UMCRC** University of Mindanao Coleoptera Research Center, Mindanao, Philippines;

**CMUUM** Central Mindanao University University Museum, Mindanao, Philippines;

**MBLI** private collection of Dr. Maurizio Bollino, Lecce, Italy.

## Results

### Metapocyrtus kitangladensis sp. n.

Taxon classificationAnimaliaColeopteraCurculionidae

http://zoobank.org/884586B9-7EAB-4BF0-932A-0C0F0D01CB3F

Figs 1–4 , 5–10 , 11–12 , 14

#### Material.

**Figures 1–4.**
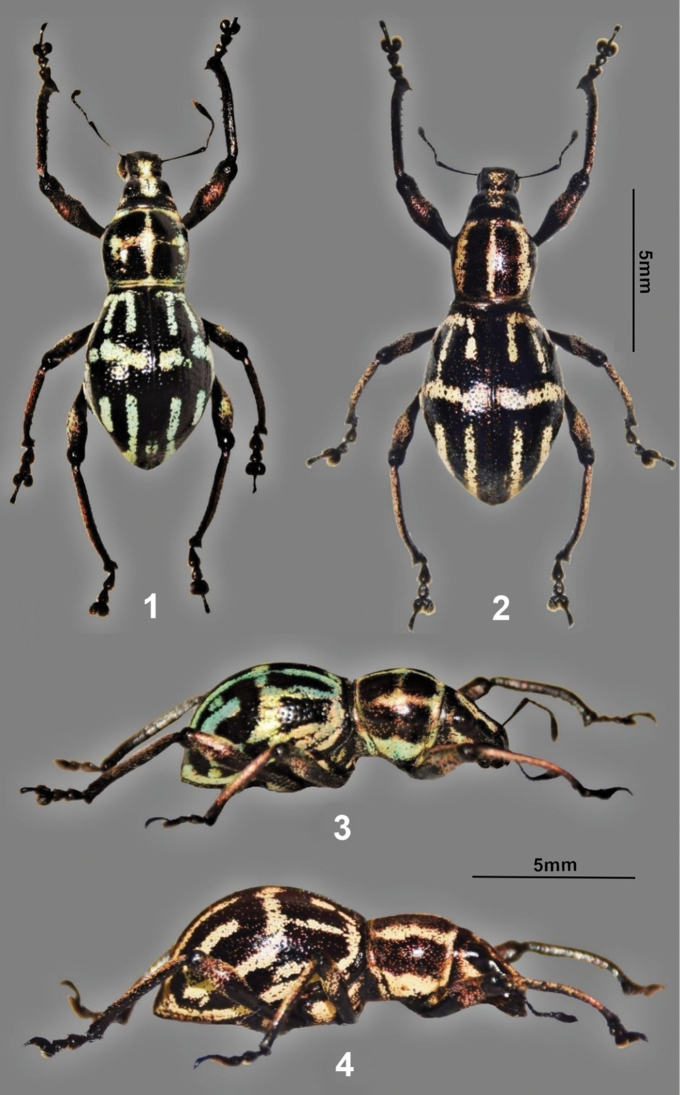
*Metapocyrtuskitangladensis* sp.n. **1** male holotype, dorsal view **2** female, dorsal view **3** ditto, lateral view **4** ditto, lateral view.

***Holotype*** (Fig. 1A, B), male: Philippines – Mindanao / Mt. Kitanglad Range Natural Park/ Bukidnon / July, 2018 / coll. Medina. Presently in UMCRC, it will be deposited in Philippine National Museum of Natural History (PNMNH) formerly Philippine National Museum (PNM). ***Paratypes.*** 3♂♂, 1 ♀: Philippines - Mindanao / Mt. Kitanglad Range Natural Park/ Bukidnon / V-VII.2018 / coll. Medina; 1 ♂: Philippines- Mindanao / Marilog District / Davao City / June, 2018 / coll. Van Dam; 1 ♀: Mindanao Marilog District / Davao City / June, 2018, presently deposited in UMCRC; 22 ♂♂, 16 ♀♀: Philippines – Mindanao / Mt. Katapagan / (Davao del Sur Province) / IX-X.2012 / coll. Bollino; 1 ♂: Philippines - Mindanao / Katapagan / (Davao del Sur Province) / IX-XI.2016 / coll. Bollino; 2 ♂♂, 1 ♀: Philippines - Mindanao / Buda Brgy. / (Davao City, Davao del Sur) / V.2017 / coll. Bollino; 4 ♂♂, 1 ♀: Philippines - Mindanao / Mt. Apo / XI 2010 / coll. Bollino, all in MBLI.

#### Diagnosis.

*Metapocyrtuskitangladensis* sp. n. is similar in general appearance to *Metapocyrtusperpulcheroides* Schultze, 1923 which was described from Kalinga Province, Luzon Island. In addition to the unique scaly markings on the pronotum and elytra of *Metapocyrtuskitangladensis* sp. n., the new species differs from *Metapocyrtusperpulcheroides* for having a subglobular pronotum, a less prominent transverse groove on rostrum, and having unique male and female genital structures.

#### Description.

Dimensions: LB: 10.5–11.5 (holotype 10.5 mm). LR: 1.5–2.0 (1.7 mm). WR: 1.4–1.7 (1.5 mm). LP: 3.5–3.8 (3.6 mm). WP: 3.9–4.0 (3.9 mm). LE: 7.5–8.1 (7.5 mm). WE: 5.2–5.6 (5.4 mm). N=5 for all measurements.

Body black; pronotum, head and legs coppery black, weakly lustrous with sparse pale yellow, green and violet scales; body surface weakly lustrous with golden yellow, orange, greenish, turquoise and bluish scales. Eyes, antennae, and tarsomeres black.

Head with the following markings: a) dense elongated pale orange and turquoise stripes under eye on each lateral side diminishing towards apex of rostrum, and b) elongated stripe of yellow, green, and orange scales from vertex to basal half of the rostrum at times confluent with lateral stripe. Rostrum rugose, longer than wide with minute light yellow setae and long yellow hairs towards the apex; dorso-apically slightly convex; prominent transverse basal groove, and longitudinal median groove forming a cross shape. Front with deep depression covered with dense scales. Eyes small and weakly convex. Antennal scape as long as the funicle plus club, with flattened hairs and sparse scales. Funicular segments I and II almost of the same length, three times longer than wide; segments III–VII as long as wide; club subellipsoidal, nearly three times longer than wide.

**Figures 5–10.**
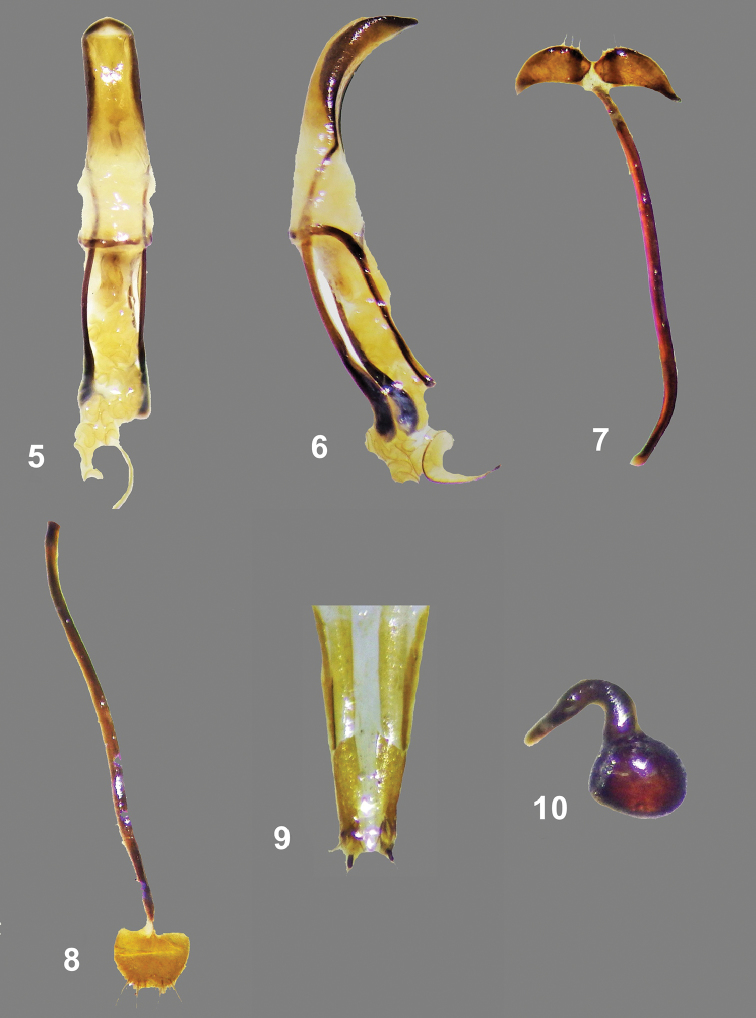
Male genitalia and female terminalia of *Metapocyrtuskitangladensis* sp. n. **5** aedeagus, ventral view **6** ditto, lateral view **7** sternite IX, dorsal view **8** sternite VIII, ventral view **9** ovipositor, dorsal view **10** spermatheca.

Pronotum subglobular, widest at middle, weakly convex, glabrous, with very minute and sparse punctures; thin strips of golden yellow to turquoise scales at the anterior, posterior and latero-ventral margin; three thin longitudinal stripes dorsally at times with transverse stripe intersecting the median stripe forming a cross.

Elytra with regular weakly striate-punctate intervals with sparse scales, moderately convex, with few short hairs. Each elytron with the following golden yellow and turquoise to light blue markings: 1) three longitudinal stripes from behind base at interval II, IV and VI which may or may not be reach median transverse stripe; stripes confluent at base; 2) stripe on lateral margin extending from base towards the apex of the elytra; 3) thin transverse band in the middle part of elytra, medially; 4) thin longitudinal stripe between interval I and II extending from middle of the elytra to apex and confluent with lateral margin stripe, may or may not be connected with median transverse stripe; 5) apical triangular stripe extending from apex of each elytron to apical third, laterally connected with median marking. Underside weakly lustrous, pubescent with pale yellow and green scales on the basal margin of the pronotum and latero-ventral side of ventrites I and II and sometimes including ventrites III and IV.

Legs with strongly clavate femora. Femora covered with short hairs and sparse scales along posterior margins. Each tibia fringed with pubescence along internal margin, sparsely mixed with short hairs. Apical part of femora with dense orange and violet scales and short setae. Tibiae with sparse scales and short setae, with toothed projections along internal edge.

Tarsomeres covered by sparse pubescence.

Male genitalia as shown in Figures 5–7.

**Figures 11, 12.**
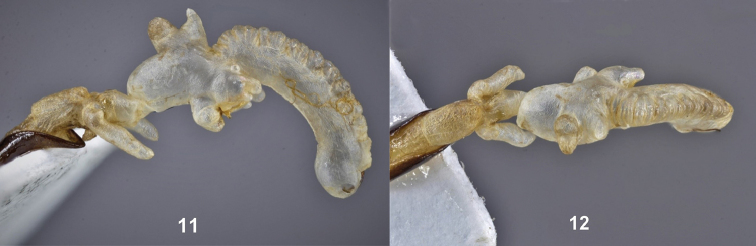
*Metapocyrtuskitangladensis* sp.n. **11** everted endophallus, lateral view **12** everted endophallus, dorsal view (photographs by Dr Maurizio Bollino).

Everted endophallus as shown in Figures 11, 12.

#### Etymology.

The new species is named after Mt Kitanglad Range Natural Park (MKRNP), the park where the holotype was collected. It is a Latinized adjective.

Notes on the ecology and distribution

**Figures 13, 14.**
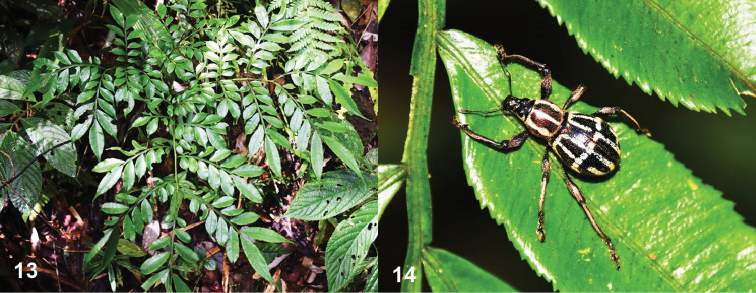
**13***Angiopterisevecta*, food plant of *M.kitangladensis* sp. n. **14***M.kitangladensis* sp. n. in its natural habitat.

*Metapocyrtuskitangladensis* sp. n. was collected in the secondary forest of Barangay Cinchona, MKRNP as well as the degraded secondary forests of Marilog District, both at around 1200 m a.s.l. The new species was mostly collected on the leaves of *Angiopterisevecta* (G.Forst.) Hoffm. (Marattiaceae) in the sloppy trail towards the forest edge (Fig. 13). It was noted that the young leaves of this fern are the main food source of this species. In Marilog District, the specimens were collected in the vegetation along the trails of Epol Falls and forest edges of Mt Malambo. All specimens collected from Marilog and MKRNP were collected in open areas often in ridges and along the streams.

*Metapocyrtuskitangladensis* sp. n. has been recorded from MKRNP, Mt Dulang-dulang, Mt Kiamo (Bukidnon), Marilog District, Barangay Buda, and Davao del Sur (Davao region) in Mindanao Island. These localities belong to Central Mindanao biogeographic region (Dickerson, 1928). Mindanao has five known biogeographic regions namely Eastern Mindanao, Central Mindanao, Western Mindanao, Southwestern and Northwestern Mindanao biogeographic regions (Dickerson, 1928). Based on collection and field observation, Bukidnon and Marilog’s Pachyrhynchini fauna shows hefty similarities. Some of the notable species recently found in Marilog District which are also found abundantly in Bukidnon are *Pachyrhynchussulphureomaculatus* Schultze, 1922, *Pachyrhynchuserichsoni* GR Waterhouse, 1841, *Pachyrhynchusspeciosus* GR Waterhouse, 1841, *Metapocyrtuslanusinus* Schultze, 1922, and *Metapocyrtusinsulanus* Schultze, 1919, among others. The trend of faunistic composition of Pachyrhynchini between Marilog and Bukidnon seem to follow this biogeographical demarcation which can be attributed to the flightless nature of these weevils with very limited dispersal capabilities. However, more data are needed to confirm this hypothesis.

### Notes on the mimicry with *Pachyrhynchuscumingii*

**Figures 15–17.**
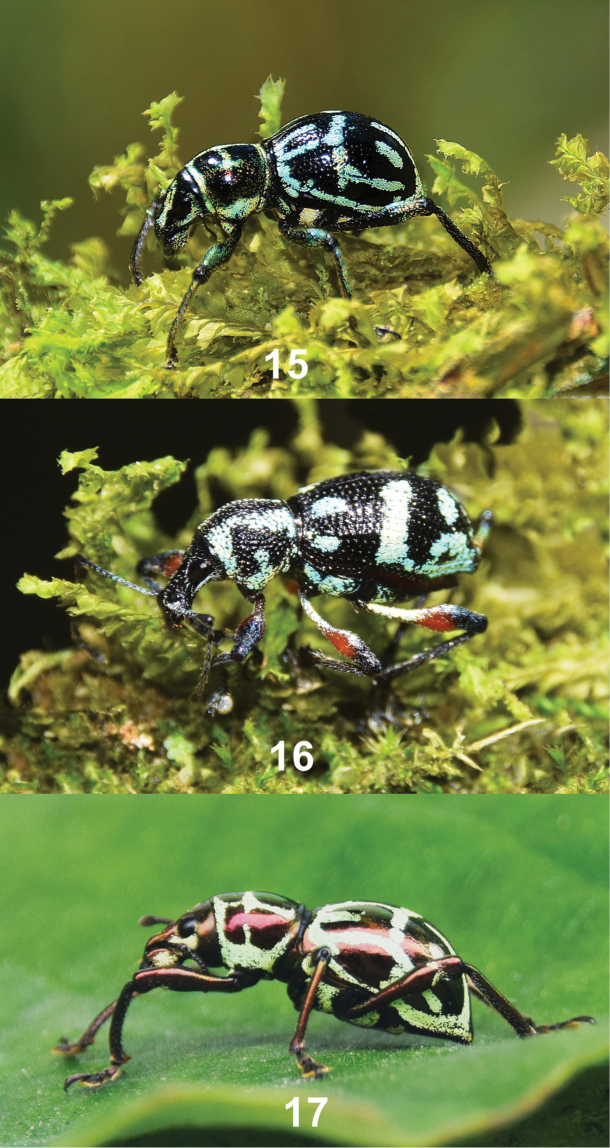
**15***Metapocyrtuskitangladensis* sp. n. **16***Coptorhynchus* sp. **17***Pachyrhynchuscumingii* GR Waterhouse, 1841.

The new species *Metapocyrtuskitangladensis* sp. n. (Fig. 15) has very similar elytral markings with *Coptorhynchus* sp. (Fig. 16) and *Pachyrhynchuscumingii* (Fig. 17), which are found in the same locality. This is the first mimic recorded for *Pachyrhynchuscumingii*. The mimicry between the three species can be considered as Mullerian since all species share similar defense mechanism which is the hardness of their elytra. This is one among the many new mimic records for Mindanao Island. *Pachyrhynchus* Germar, 1823, *Metapocyrtus* and several other weevils has been known to exhibit such mimicry, but it has been barely studied and many new mimics awaits discovery.

### Key to the *Pachyrhynchuscumingii* mimicry complex

**Table:** 

1	Rostrum distinctly elongate, obviously longer than frons, antennal scrobes laterally oriented	***Coptorhynchus* sp.**
–	Rostrum of medium length, antennal scrobes laterally curving downwards in front of eyes at sides of rostrum	**2**
2	Head without a distinct transverse groove between frons and rostrum, apical half of rostrum dorsally swollen, and antennal scape not reaching hind margin of eye	***Pachyrhynchuscumingii***
–	Head with a distinct transverse groove between forehead and rostrum, Apical half of rostrum not swollen dorsally, and antennal scape reaching beyond the hind margin of eye	***Metapocyrtuskitangladensis* sp. n.**

## Supplementary Material

XML Treatment for Metapocyrtus kitangladensis
